# A study of the crystal structures, supra­molecular patterns and Hirshfeld surfaces of bromide salts of hypoxanthine and xanthine

**DOI:** 10.1107/S2056989022005278

**Published:** 2022-05-20

**Authors:** Udhayasuriyan Sathya, Jeyaraman Selvaraj Nirmalram, Sundaramoorthy Gomathi, Durairaj Dhivya, Samson Jegan Jennifer, Ibrahim Abdul Razak

**Affiliations:** a Centre for Research and Development, PRIST Deemed to be University, Thanjavur, 613 403, Tamil Nadu, India; b Department of Chemistry, Periyar Maniammai Institute of Science and Technology, Thanjavur 613 403, Tamil Nadu, India; c X-ray Crystallography Unit, School of Physics, University Sains Malaysia, 11800, USM, Penang, Malaysia; Universidad Nacional Autónoma de México, México

**Keywords:** crystal structure, hydrogen bonding, hirshfeld surface analysis, hypoxanthine, xanthine

## Abstract

Two new crystalline salts, namely, hypoxanthinium bromide monohydrate, C_5_H_5_N_4_O^+^·Br^−^·H_2_O (**I**) and xanthinium bromide monohydrate, C_5_H_5_N_4_O_2_^+^·Br^−^·H_2_O (**II**), were synthesized and characterized by single-crystal X-ray diffraction technique and Hirshfeld surface analysis. The hypoxanthinium and xanthinium cations in salts **I** and **II** are both in the oxo-N(9)–H tautomeric form. The crystal packing of the two salts is governed predominantly by N–H⋯O, N–H⋯Br, C–H⋯Br and O–H⋯Br inter­actions described by *R* ^2^_3_(9) and *R* ^2^_2_(8) synthons. The crystal packing is also consolidated by carbon­yl⋯π inter­actions between symmetry-related hypoxanthinium (**HX^+^**) cations in salt **I** and xanthinium cations (**XA^+^**) in salt **II**. The combination of all these inter­actions leads to the formation of wave- and staircase-like architectures in salts **I** and **II**, respectively. The largest contributions to the overall Hirshfeld surface are from Br⋯H/H⋯Br contacts (22.3% in **I** and 25.4% in **II**) .

## Abstract 2

Two new crystalline salts, namely, hypoxanthinium bromide monohydrate, C_5_H_5_N_4_O^+^·Br^−^·H_2_O (**I**) and xanthinium bromide monohydrate, C_5_H_5_N_4_O_2_^+^·Br^−^·H_2_O (**II**), were synthesized and characterized by single-crystal X-ray diffraction technique and Hirshfeld surface analysis. The hypoxanthinium and xanthinium cations in salts **I** and **II** are both in the oxo-N(9)–H tautomeric form. The crystal packing of the two salts is governed predominantly by N—H⋯O, N—H⋯Br, C—H⋯Br and O—H⋯Br inter­actions described by 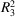 (9) and 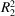 (8) synthons.

## 1. Chemical context

Over the past several decades, non-covalent inter­actions have been found to play a prominent role in coordination chemistry, materials science and pharmaceutical science (Černý & Hobza, 2007 ▸; Desiraju, 2013 ▸; Perumalla & Sun, 2014 ▸). Understanding the role of non-covalent inter­actions is important in the context of crystal engineering (Aakeröy *et al.*, 2010 ▸; Pogoda *et al.*, 2018 ▸; Cavallo *et al.*, 2016 ▸; Desiraju *et al.*, 2013 ▸) in order to design solids with desired properties. When it comes to pharmaceutics, active pharmaceutical ingredients (APIs) are known to exist in different solid forms such as salts, co-crystals, solvates, polymorphs and amorphous solids (Aaltonen *et al.*, 2009 ▸). The salt and co-crystal forms of APIs have improved their solubility and bioavailability when compared to pure APIs (Thackaberry, 2012 ▸; Xu, *et al.*, 2014 ▸). Drugs with low solubility/bioavailability are usually converted to their salts or crystallized in their co-crystal/polymorphic/solvate forms to enhance their properties. Herein, we report two new salts of hypoxanthine (**HX**) and xanthine (**XA**).

Hypoxanthine (C_5_H_4_N_4_O) [systematic name: 1,9-di­hydro-purine-6-one] and xanthine (C_5_H_4_N_4_O_2_) [systematic name: 3,7-di­hydro-purine-2,6-dione] are well-known purine-based nucleotides (Emel’yanenko *et al.*, 2017 ▸) present in *t*-RNA and DNA in the form of the nucleoside inosine (Plekan *et al.*, 2012 ▸). Purine derivatives are widely known for their therapeutic applications such as antagonization of the adenosine receptor, anti-inflammatory, anti­microbial, anti­oxidant, anti-tumour, anti-asthmatic and psycho-stimulant drug activity (Meskini *et al.*, 1994 ▸; Burbiel *et al.*, 2006 ▸). **HX** and **XA** are also found as inter­mediates in the biological degradation of nucleic acid to uric acid. Furthermore, **HX** is used as an indicator of hypoxia and it is known to inhibit the effect of several drugs (Dubler *et al.*, 1987*a* ▸,*b* ▸). It is also used to destroy harmful agents such as cancer cells (Susithra *et al.*, 2018 ▸). Purine-based derivatives of **HX** and **XA** bind with the DNA base pairs through weak hydrogen bonds (Latosińska *et al.*, 2014 ▸; Rutledge *et al.*, 2007 ▸). Additionally, hypoxanthine-guanine phospho­ribosyl transferase plays an important role in activating anti­viral drugs in the human body and xanthine has been used as a mild stimulant drug (Faheem *et al.*, 2020 ▸).

The structure of hypoxanthine and xanthine consists of fused six-membered pyrimidine and five-membered imidazole rings. **HX** and **XA** can exist in two tautomeric forms, oxo-N(7)–H and oxo-N(9)–H (Plekan *et al.*, 2012 ▸; Gulevskaya & Pozharskii, 1991 ▸), as shown below. So far, two polymorphic forms of **HX** (Schmalle *et al.*, 1988 ▸; Yang & Xie, 2007 ▸) and a limited number of hypoxanthinium and xanthinium salts have been reported in the literature; hypoxanthinium nitrate monohydrate, hypoxanthinium chloride monohydrate (Cabaj *et al.*, 2019 ▸; Schmalle *et al.*, 1990 ▸; Sletten & Jensen, 1969 ▸), xanthinium nitrate monohydrate and xanthinium hydrogensulfate monohydrate (Sridhar, 2011 ▸). 
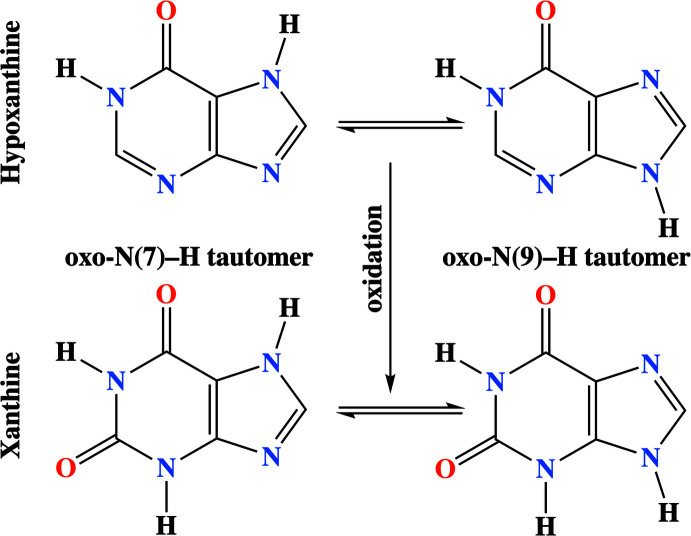


In the hypoxanthinium salts, the hypoxanthine mol­ecule is usually also protonated at the N7 position, resulting in the oxo-N(9)–H tautomer. Similarly, xanthinium nitrate monohydrate, xanthinium hydrogensulfate monohydrate (Sridhar, 2011 ▸) and xanthinium perchlorate dihydrate (Biradha *et al.*, 2010 ▸) are also in the oxo-N(9)–H tautomeric form and are therefore protonated on the N7 position. Studies of non-covalent inter­actions involving hypoxanthine and xanthine bases with inorganic acids have increased because their hydrogen-bonding patterns are similar to those of purine bases (Maixner & Zachova, 1991 ▸; Sridhar, 2011 ▸; Kistenmancher & Shigematsu, 1974 ▸). In the current work, the crystal structures, supra­molecular packing patterns and Hirshfeld surface analyses of hypoxanthinium bromide monohydrate (**I**) and xanthinium bromide monohydrate (**II**) are reported. 
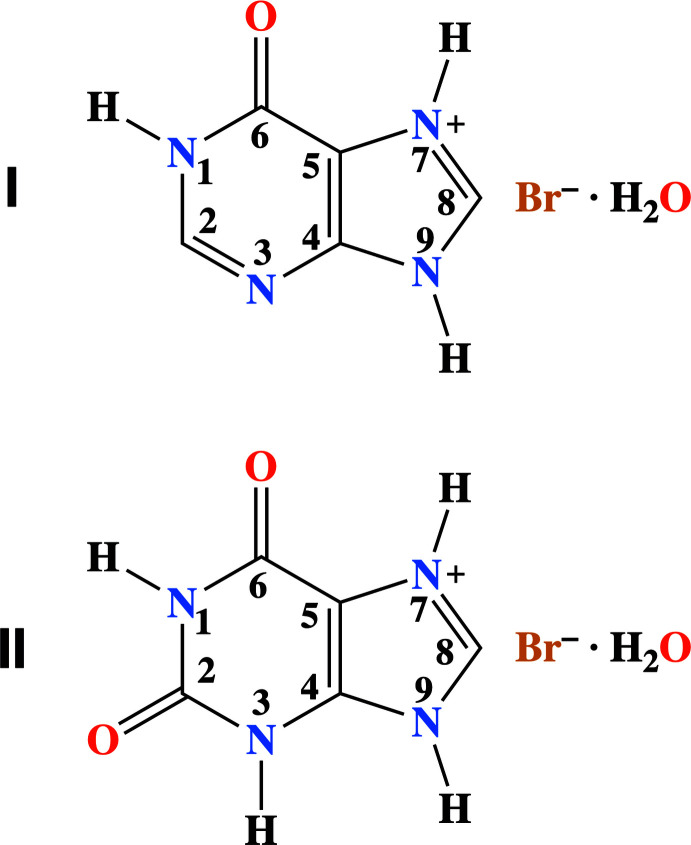


## 2. Structural commentary

**Figure 1:**
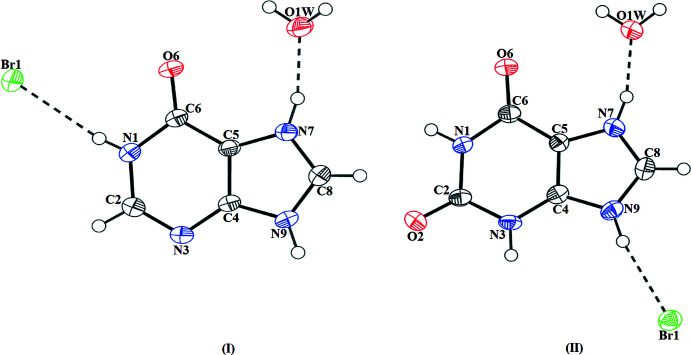
*ORTEP* view of the mol­ecular components of salts **I** and **II**, showing the atom-labelling scheme. Displacement ellipsoids are drawn at 50% probability level. Hydrogen bonds are shown as dashed lines.

Hypoxanthinium bromide monohydrate (**I**) crystallizes in the monoclinic space group *P*2_1_/*c* with one hypoxanthinium cation (**HX^+^**), one bromide anion (**Br^−^**) and one water mol­ecule in the asymmetric unit, as shown in Fig. 1 ▸. Here, the **HX^+^** cation exists in the oxo-N(9)–H tautomeric form with the N7 atom of the purine ring protonated, as can be seen from the N—C bond distance [N7—C8 = 1.3219 (17) Å *vs* N9—C8 = 1.3419 (18) Å] and C—N—C bond angles [C5—N7—C8 = 107.98 (11)° and C4—N9—C8 = 108.32 (10)°]. Those values are similar to those in the crystal structure of hypoxanthinium chloride monohydrate [N7—C8 = 1.325 (2) Å and N9—C8 = 1.336 (2) Å, C5—N7—C8 = 107.35 (16)° and C4—N9—(C8 = 108.28 (15)°; Kalyanaraman *et al.*, 2007 ▸; Sletten & Jensen, 1969 ▸]. The N3—C4—C5—N7 and N9—C4—C5—C6 torsion angles are 179.07 (12) and −179.58 (12)°, respectively. These values are similar to those observed in the crystal structure of the neutral hypoxanthine mol­ecule (Schmalle *et al.*, 1988 ▸; Yang & Xie, 2007 ▸). The **HX^+^** cation, **Br^−^** anion and the water mol­ecule inter­act through N—H⋯Br, N—H⋯O and C—H⋯Br hydrogen bonds with donor–acceptor distances N⋯Br = 3.2419 (13) Å, N9⋯O6 = 2.7579 (14) Å and C8⋯Br1 = 3.4875 (15) Å (Table 1 ▸), forming an 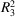 (9) motif. The water mol­ecule present in the lattice prevents the formation of base pairs (Varani & McClain, 2000 ▸) between the **HX^+^** cations.

Xanthinium bromide monohydrate (**II**) also crystallizes in the monoclinic space group *P*2_1_/*c* with one xanthinium cation (**XA^+^**), one bromide anion (**Br^−^**) and one water mol­ecule in the asymmetric unit (Fig. 1 ▸). The **XA^+^** cation has the N7—C8 bond [1.312 (5) Å] shorter than N9–C8 one [1.344 (5) Å]. The C—N—C bond angles are C5—N7—C8 = 108.2 (3)° and C4—N9—C8 = 107.7 (3)° and, therefore, the cation can also be described as the oxo-N(9)–H tautomer. These values are similar to those in xanthinium perchlorate dihydrate [N7—C8 = 1.314 (3) Å, N9—C8 = 1.341 (3) Å, C5—N7—C8 = 108.3 (16)° and C4—N9—C8 = 107.58 (15)°; Biradha *et al.*, 2010 ▸). The N3—C4—C5—N7 and N9—C4—C5—C6 torsion angles in **II** are 179.07 (12)° and −179.58 (12)°, respectively. Finally, the two symmetry-related **XA^+^** cations in **II** form a base pair similar to that observed between guanine and uracil (Varani & McClain, 2000 ▸).

## 3. Supra­molecular features

**Figure 2:**
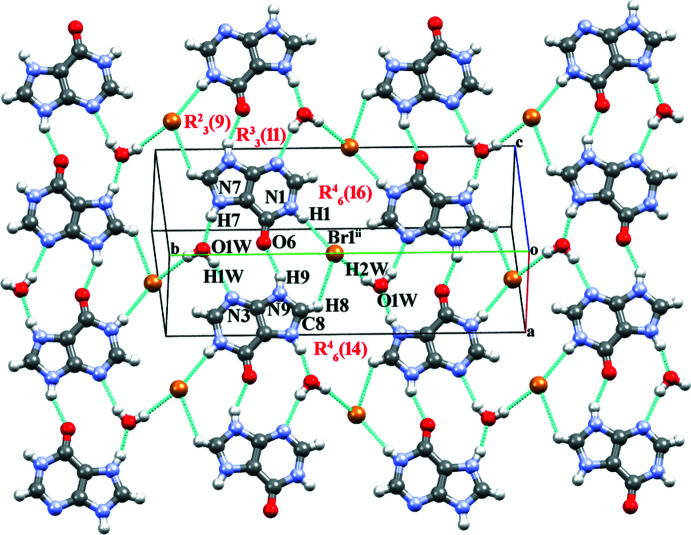
Hypoxanthinium and bromide ions in salt **I** forming ribbons together with water mol­ecules through O—H⋯Br, N—H⋯Br and C—H⋯Br inter­action. [Symmetry codes: (i) −1 − *x*, − 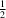 + *y*, 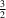 − *z*; (ii) 1 + *x*, 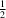 − *y*, − 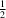 + *z*; (iii) −*x*, 1 − *y*, 1 − *z*].

**Figure 3:**
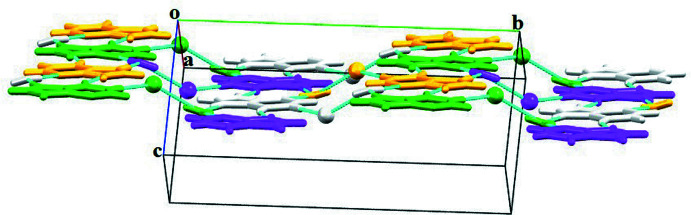
A view of three-dimensional wave-like supra­molecular architecture along the *b*-axis direction.

**Figure 4:**
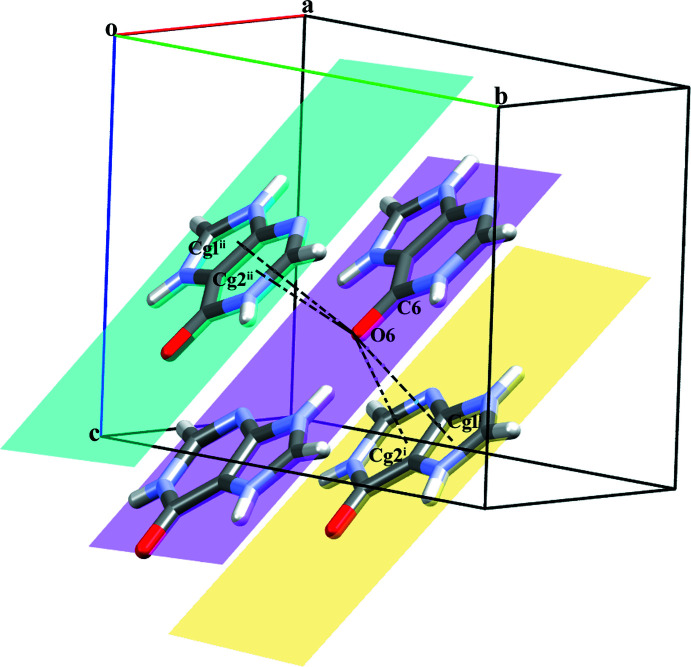
A view of the C=O(carbon­yl)⋯π inter­actions (dashed lines) between the **HX^+^** cations in salt **I**. [Symmetry codes: (i) −1 + *x*, *y*, *z*; (ii) *x*, 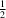 − *y*, 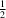 + *z*].

In **I**, the protonated **HX^+^** cation inter­acts with another inversion-related **HX^+^** and **Br^−^** pair *via* N1—H1⋯Br1, C8—H8⋯Br1^ii^ and N9—H9⋯O6^ii^ hydrogen bonds (Table 1 ▸). These inter­actions lead to the formation of a nine-membered ring with 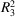 (9) (type ***D*** [Chem scheme3]) primary graph-set motif (Sletten & Jensen, 1969 ▸). Along with this, the **HX^+^** cation inter­acts with another inversion-related **HX^+^** cation and a water mol­ecule through O1*W*—H1*W*⋯N3^iii^ and N7—H7⋯O1*W* ^ii^ hydrogen bonds. The combination of these inter­actions leads to the formation of an eleven-membered 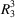 (11) (type ***I*** [Chem scheme3]) ring motif. The inter­action is very similar to the water-mediated base pairs observed in the crystal structure of hypoxanthinium chloride and the nucleobase pairs in DNA and RNA (Sletten & Jensen, 1969 ▸; Reddy *et al.*, 2001 ▸; Brandl *et al.*, 2000 ▸). Here the O1*W* atom of the water mol­ecule acts as both a hydrogen-bond donor and a hydrogen-bond acceptor. The 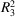 (9) and 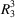 (11) ring motifs combine to form a supra­molecular ribbon. Adjacent ribbons are connected through pairs of O1*W*—H2*W*⋯Br1 hydrogen bonds with 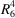 (16) and 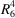 (14) (types ***N*** and ***O*** motifs[Chem scheme3]) ring motifs, respectively, through pairs of C8—H8⋯Br1^i^ and N7—H7⋯O1*W* ^ii^ hydrogen bonds (Fig. 2 ▸). The combination of all these inter­actions leads to the formation of a wave-like supra­molecular architecture that extends along the *b*-axis direction (Fig. 3 ▸). The crystal structure is further consolidated by carbon­yl⋯π inter­actions (C6=O6 and π cloud of the imidazole (centroid *Cg*1) and pyridine (centroid *Cg*2) rings of the **HX^+^** cation) between symmetry-related cations with C=O⋯*Cg*1^iv^, C=O⋯*Cg*1^v^, C=O⋯*Cg*2^iv^ and C=O⋯*Cg*2^v^ distances of 3.5796 (12), 3.2478 (12) Å, 3.3862 (12) and 3.4747 (12) Å, respectively, and angles of 101.58 (8), 91.45 (8), 105.03 (8) and 103.46 (8)**°**, respectively [symmetry codes: (iv) −1 + *x*, *y*, *z*; (v) *x*, 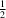 − *y*, 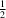 + *z*] (Fig. 4 ▸). Salt **I** is isomorphous with hypoxanthinium chloride monohydrate (Sletten & Jensen, 1969 ▸). 
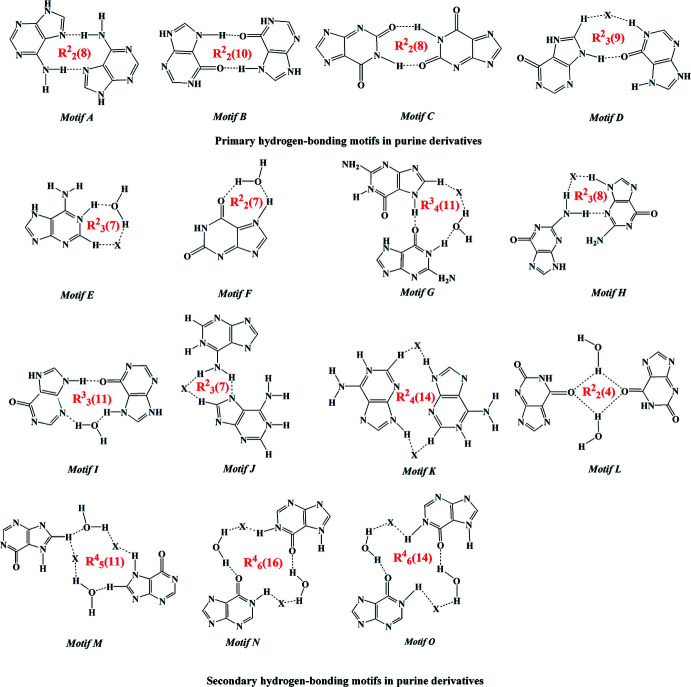


**Figure 5:**
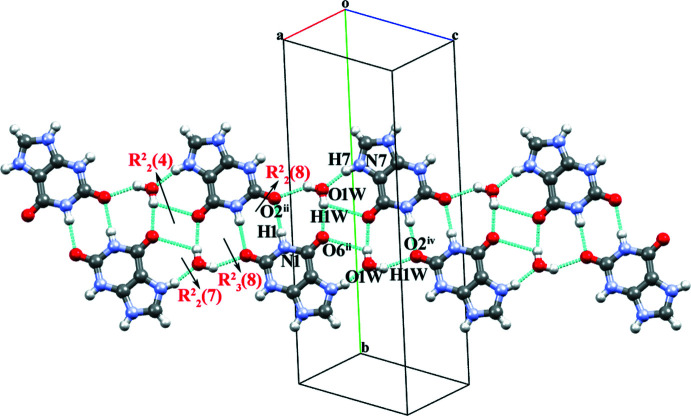
Formation of a supra­molecular ribbon with a *DADA* array in salt **II** *via* N—H⋯O and O—H⋯O hydrogen bonds between cations and water mol­ecules.

**Figure 6:**
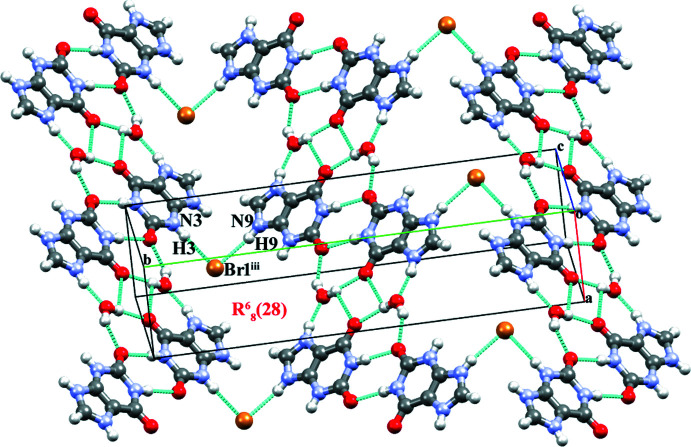
Supra­molecular ribbons connecting adjacent ribbons through N—H⋯Br inter­actions. [Symmetry codes: (i) 1 − *x*, 1 − *y*, 2 − *z*; (ii) 2 − *x*, 1 − *y*, 1 − *z*; (iii) *x*, 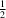 − *y*, 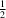 + *z*; (iv) 1 + *x*, *y*, −1 + *z*].

**Figure 8:**
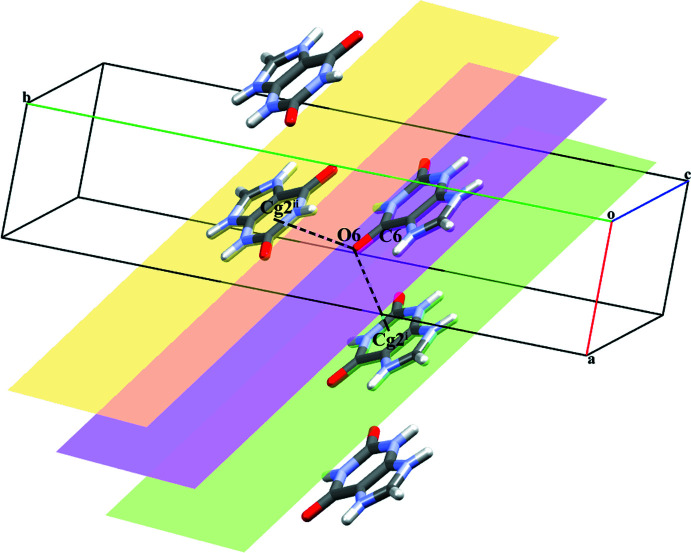
A view of the C=O(carbon­yl)⋯π inter­actions between **XA^+^** cations in salt **II**. [Symmetry codes: (i) 1 + *x*, *y*, *z*; (ii) 1 − *x*, 1 − *y*, 1 − *z*].

In the crystal structure of salt **II**, the **XA^+^** cation inter­acts with its inversion-related equivalent to form a dimer through a pair of N1—H1⋯O2^i^ hydrogen bonds (Table 2 ▸) with an 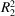 (8) graph-set motif (type ***C*** in the scheme above). The dimer is flanked on both sides by a water mol­ecule (O1*W*), forming a pair of O1*W*—H2*W*⋯O2^iv^ and O1*W*—H1*W*⋯O6^ii^ hydrogen bonds with an 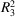 (8) graph-set motif (type ***H*** [Chem scheme3]), leading to the formation of a tetra­meric unit. The tetra­meric unit is formed by an alternate arrangement of 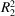 (8) and 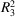 (8) ring motifs, which extend as *DADA* array (dimeric units held together by four hydrogen bonds between the self-complementary *DADA* arrays; *D* = donor and *A* = acceptor) along the *ac* plane. Neighbouring tetra­meric units are then connected through two sets of 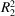 (7) motifs (Jeffrey & Saenger, 1991 ▸) formed by N7—H7⋯O1*W* and O1*W*—H1*W*⋯O6^ii^ hydrogen bonds and an 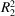 (4) (type ***L***) motif formed by a pair of O1*W*—H1*W*⋯O6^ii^ inter­actions. The tetra­meric units combine into a supra­molecular ribbon extended along the *ac* plane (Fig. 5 ▸). Neighbouring perpendicular supra­molecular ribbons are then inter­connected through pairs of N3—H3⋯Br1^iii^ and N9—H9⋯Br1 hydrogen bonds with an 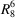 (28) ring motif, which assembles them into a staircase-like supra­molecular architecture as shown in Figs. 6 ▸ and 7 ▸. The crystal structure is further consolidated by carbon­yl⋯π inter­actions between symmetry-related **XA^+^** cations [C6=O6 and π cloud of the pyridine ring (centroid *Cg*2) of the **XA^+^** unit) with C=O⋯*Cg*2^vi^ and C=O⋯*Cg*2^vii^ distances of 3.366 (3) and 3.477 (3) Å, respectively, and angles of 108.2 (2) and 118.7 (2)**°** [symmetry codes: (vi) 1 + *x*, *y*, *z*; (vii) 1 − *x*, 1 − *y*, 1 − *z*; Fig. 8 ▸).

## 4. Hirshfeld surface analysis

**Figure 9:**
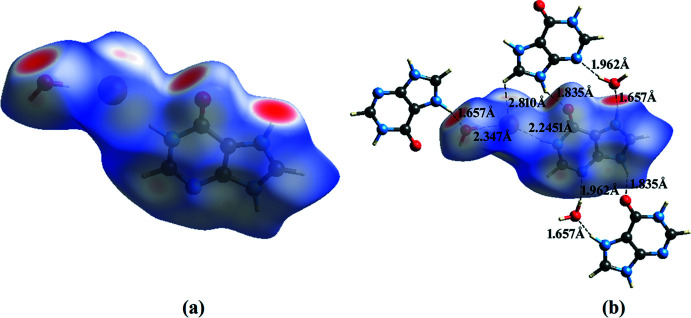
(*a*) Hirshfeld surface mapped over *d*_norm_ for salt **I**. (*b*) Inter­molecular inter­actions and the three-dimensional Hirshfeld surface for salt **I**.

**Figure 11:**
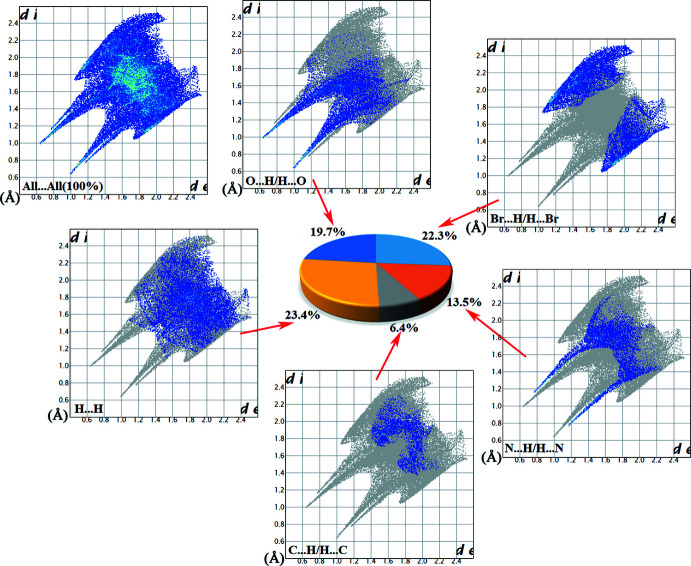
Hirshfeld surface analysis and two-dimensional fingerprint plots for salt **I** plotted over *d*_norm_, with inter­actions to neighbouring fragments shown as dashed lines.

Hirshfeld surface analyses and their associated two-dimensional fingerprint plots (McKinnon *et al.*, 2007 ▸; Spackman & Jayatilaka, 2009 ▸) were generated using *Crystal Explorer 17.5* (Turner *et al.*, 2017 ▸). The Hirshfeld surfaces of the title compounds mapped over *d*_norm_ feature several red spots in the regions of *D*–*A* (*D* = donor, *A* = acceptor) inter­actions (Cárdenas-Valenzuela *et al.*, 2018 ▸; Atioğlu *et al.*, 2018 ▸). In this regard, the contribution of the inter­atomic contacts to the *d*_norm_ surface map can help differentiate whether the contact is longer (blue) or shorter (red) than the sum of the van der Waals radii of the two inter­acting atoms. The Hirshfeld surfaces of salts **I** and **II** are shown in Fig. 9 ▸ *a* and 10*a* ▸, respectively and the hydrogen-bonding inter­actions between the hydrated ion pairs **I** and **II** and the respective neighbouring moieties are shown in Fig. 9 ▸ *b* and 10*b* ▸, respectively. The intense red spots on the Hirshfeld surface indicate the shortest inter­atomic distances corresponding to the hydrogen bonds. They are also clearly identified by the two long spikes in the fingerprint plots and can be qu­anti­fied using the percentage distribution of the inter­acting types. Such analyses of the salts **I** and **II** are shown in Figs. 11 ▸ and 12 ▸ giving the following contributions: All (100%), O⋯H/H⋯O (**I** 19.7%, **II** 23.4%), N⋯H/H⋯N (**I** 13.5%, **II** 7.5%) C⋯H/H⋯C (**I** 6.4%, **II** 9.6%), H⋯H/H⋯H (**I** 23.4%, **II** 15.9%) and C⋯C/C⋯C (**I** 0.9%, **II** 0.1%) (Table 5), indicating that the most abundant contact is Br⋯H/H⋯Br with 22.3% in **I** and 25.4% in **II**, respectively.

## 5. Comparative analysis

The data obtained by comparative analysis of the crystal structures, supra­molecular inter­actions, hydrogen-bonding motifs and packing patterns of structurally similar halide salts such as adeninium bromide, adeninium chloride, guaninium bromide, guaninium chloride and hypoxanthinium chloride (Maixner & Zachova, 1991 ▸; Sridhar, 2011 ▸; Kistenmancher & Shigematsu, 1974 ▸; Langer & Huml, 1978 ▸) are listed and compared in Table 3 ▸.

Salt **I** has similar unit-cell parameters and packing patterns to the hypoxanthinium chloride salt. The mol­ecular recog­nition between the hypoxanthine base and acid happens *via* N—H⋯O, C—H⋯Br/Cl and N—H⋯Br/Cl hydrogen-bond motifs with 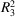 (9) (type ***D*** [Chem scheme3]), 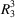 (11) (type ***I*** [Chem scheme3]), 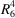 (16) (type ***N*** [Chem scheme3]) and 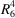 (14) (type ***O*** [Chem scheme3]) graph-set motifs. Salt **II** forms base pairs *via* N—H⋯O hydrogen bonds described by 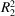 (8) (type ***C*** [Chem scheme3]), 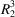 (8) (type ***H*** [Chem scheme3]) (Wei, 1977 ▸; Maixner & Zachova, 1991 ▸), 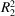 (7) (type ***F*** [Chem scheme3]) and 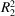 (4) (type ***L*** [Chem scheme3]) graph-set motifs. Salt **II** cannot be compared with its chloride analogue since its crystal structure has not yet been reported.

A comparison between some related purine-based chloride and bromide salts revealed that type ***A*** [Chem scheme3], ***B*** [Chem scheme3] and ***C*** [Chem scheme3] hydrogen-bond motifs are predominant. The commonly observed motifs in purine based salts are shown in the scheme. A comparison of salts **I** and **II** with the reported crystal structures revealed that the bromide and chloride salts of **I** are isomorphous and therefore, one might predict, the unreported xanthinium chloride monohydrate could be isomorphous with its bromide salt **II**.

## 6. Database survey

A survey of the Cambridge Structural Database (CSD, version 5.43, update of March 2022; Groom *et al.*, 2016 ▸) for reported structures of hypoxanthine and xanthine derivatives identified the hypoxanthine mol­ecule (CSD refcodes GEBTUC and GETBUC01; Schmalle *et al.*, 1988 ▸; Yang & Xie, 2007 ▸) and the following salts: hypoxanthinium nitrate monohydrate (BONKOE and BONKOE54; Cabaj *et al.*, 2019 ▸; Schmalle *et al.*, 1990 ▸), hypoxanthinium chloride monohydrate (HYPXCL and HYPXCL01; Sletten & Jensen, 1969 ▸; Kalyanaraman *et al.*, 2007 ▸) as well as three xanthine salts, *viz.* xanthinium perchlorate monohydrate (VURMUR; Biradha *et al.*, 2010 ▸), xanthinium nitrate monohydrate (YADJAQ; Sridhar, 2011 ▸) and xanthinium hydrogensulfate monohydrate (YADJEU; Sridhar, 2011 ▸). In all of the hypoxanthinium salts, the hypoxanthine mol­ecule is protonated at the N7 position and inter­acts with the anion through N—H⋯Cl/O and C=O⋯π inter­actions. In the xanthinium salts, the xanthine mol­ecules are protonated at the N7 position in xanthinium nitrate monohydrate and xanthinium hydrogensulfate monohydrate and at the N9 position in xanthinium perchlorate monohydrate. In all of the crystal structures, the xanthinium cation inter­acts with the anion through N—H⋯O, O—H⋯O and C=O⋯π inter­actions.

## 7. Synthesis and crystallization

A general method was used for the preparation and crystallization of the hypoxanthinium bromide monohydrate (**I**) and xanthinium bromide monohydrate (**II**) using the following qu­anti­ties: 0.0340 mg (0.25mmol) of hypoxanthine for **I** and 0.0380 mg (0.25 mmol) of xanthine for **II**.

The indicated amount of the base was dissolved in 20 mL of distilled water and 2 mL of hydro­bromic acid (5% in water) were added. The reaction mixture was heated to 358 K for 30 min using a water bath. The resulting solution was allowed to slowly evaporate at room temperature. After a few days, colourless plate-like crystals were obtained.

## 8. Refinement

Crystal data, data collection and structure refinement details for salts **I** and **II** are summarized in Table 4 ▸. All C-bound hydrogen atoms were placed in idealized positions and refined using a riding model, with C—H = 0.93 Å and *U*_iso_(H) = 1.2*U*_eq_ (C). The H atoms of the water mol­ecule were located in a difference-Fourier map and refined with the O—H distance restrained to 0.85–0.86 Å and with *U*_iso_(H) = 1.5 *U*_eq_(O). The hydrogen atoms bound to the nitro­gen atoms in salts **I** and **II** were located in difference-Fourier maps and either refined freely (in **I**) or with the distance restraint N—H = 0.82 Å and with *U*_iso_(H) = 1.2*U*_eq_(N) (in **II**).

## Supplementary Material

Crystal structure: contains datablock(s) I, II. DOI: 10.1107/S2056989022005278/jq2017sup1.cif

Structure factors: contains datablock(s) I. DOI: 10.1107/S2056989022005278/jq2017Isup2.hkl

Supporting information file. DOI: 10.1107/S2056989022005278/jq2017Isup4.cml

Structure factors: contains datablock(s) II. DOI: 10.1107/S2056989022005278/jq2017IIsup3.hkl

Supporting information file. DOI: 10.1107/S2056989022005278/jq2017IIsup5.cml

CCDC references: 2170923, 2170922

Additional supporting information:  crystallographic information; 3D view; checkCIF report

## Figures and Tables

**Figure 7:**
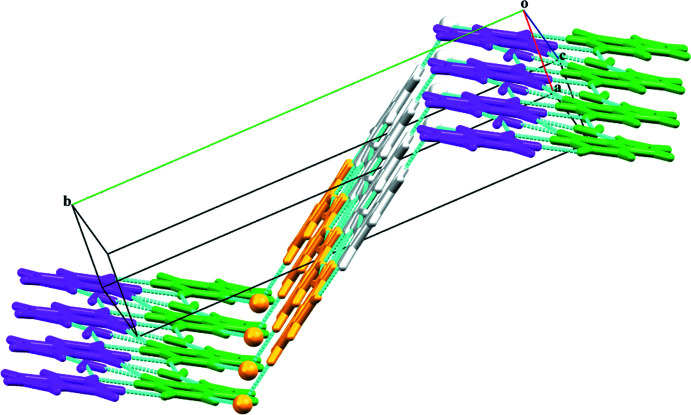
The formation of a three-dimensional supra­molecular staircase structure along the *ac* plane.

**Figure 10:**
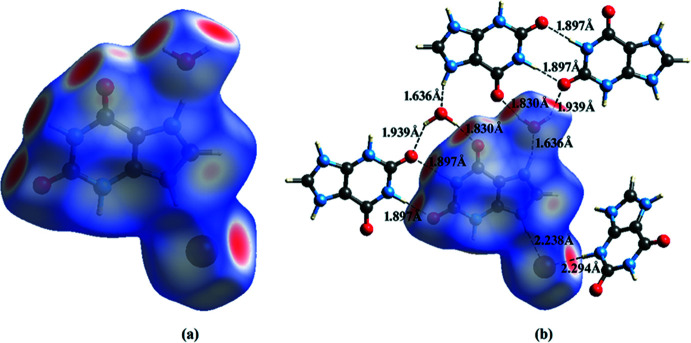
(*a*) Hirshfeld surface mapped over *d*_norm_ for salt **II**. (*b*) Inter­molecular inter­actions and the three-dimensional Hirshfeld surface for salt **II**.

**Figure 12:**
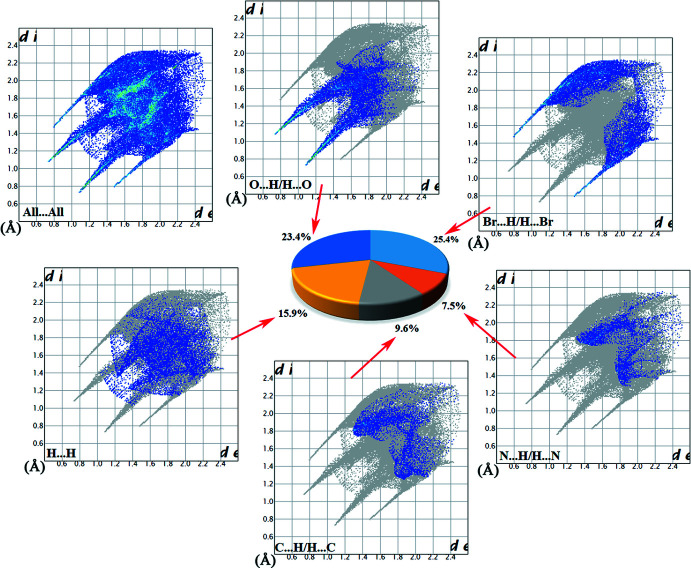
Hirshfeld surface analysis and two-dimensional fingerprint plots for salt **II** plotted over *d*_norm_, with inter­actions to neighbouring fragments shown as dashed lines.

**Table 1:** Hydrogen-bond geometry (Å, °) for **I** [Chem scheme1]

*D*—H⋯*A*	*D*—H	H⋯*A*	*D*⋯*A*	*D*—H⋯*A*
N9—H9⋯Br1^i^	0.85 (1)	3.08 (2)	3.5397 (12)	117 (2)
N9—H9⋯O6^i^	0.85 (1)	1.98 (2)	2.7579 (14)	153 (2)
N1—H1⋯Br1	0.84 (1)	2.41 (1)	3.2419 (12)	170 (2)
N7—H7⋯O1*W* ^ii^	0.85 (1)	1.81 (2)	2.6401 (16)	165 (2)
O1*W*—H1*W*⋯N3^iii^	0.86 (1)	2.08 (1)	2.9200 (16)	165 (2)
O1*W*—H2*W*⋯Br1	0.85 (1)	2.48 (1)	3.2894 (12)	161 (2)
C8—H8⋯Br1^i^	0.93	2.89	3.4875 (15)	123

Symmetry codes: (i) 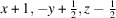 ; (ii) 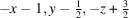 ; (iii) 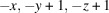 .

**Table 2:** Hydrogen-bond geometry (Å, °) for **II** [Chem scheme1]

*D*—H⋯*A*	*D*—H	H⋯*A*	*D*⋯*A*	*D*—H⋯*A*
N1—H1⋯O2^i^	0.82 (2)	2.09 (2)	2.903 (4)	175 (4)
N3—H3⋯Br1^ii^	0.82 (2)	2.48 (2)	3.301 (3)	176 (4)
N7—H7⋯O1*W*	0.82 (2)	1.81 (2)	2.609 (4)	163 (4)
N9—H9⋯Br1	0.82 (2)	2.43 (2)	3.237 (3)	172 (4)
O1*W*—H1*WA*⋯O6^iii^	0.86 (1)	1.95 (1)	2.802 (4)	171 (5)
O1*W*—H1*WB*⋯Br1^iv^	0.86 (1)	3.03 (4)	3.490 (3)	115 (3)
O1*W*—H1*WB*⋯O2^v^	0.86 (1)	2.05 (3)	2.816 (4)	149 (4)

Symmetry codes: (i) 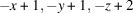 ; (ii) 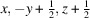 ; (iii) 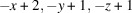 ; (iv) 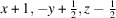 ; (v) 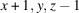 .

**Table 3:** Comparison of purine derivatives with hydro­bromic acid and hydro­chloric acid

	Adeninium bromide hemihydrate	Adeninium chloride monohydrate	Guaninium chloride monohydrate	Guaninium bromide monohydrate	Hypoxanthinium chloride monohydrate	Hypoxanthinium bromide monohydrate (**I**)	Xanthinium bromide monohydrate (**II**)
Cell parameters (*a*, *b*, *c*, β; Å, °)	9.018 (2), 4.845 (2), 19.693 (5), 112.8	8.771 (2), 4.834 (2), 19.46 (1), 114.25	4.591 (1), 9.886 (2), 18.985 (1), 99.62	4.8708 (7), 13.237 (3), 14.638 (2), 93.906 (10)	4.8295 (9), 17.7285 (22), 9.0077 (21), 94.59 (3)	4.8487 (4), 18.4455 (15), 9.0782 (7), 94.808 (1)	4.9225 (2), 22.7572 (17), 7.5601 (5) 103.003 (3)
Crystal system	Monoclinic	Monoclinic	Monoclinic	Monoclinic	Monoclinic	Monoclinic	Monoclinic
Space group	*P*2/*c*	*P*2/*c*	*P*2_1_/*c*	*P*2_1_/*c*	*P*2_1_/*c*	*P*2_1_/*c*	*P*2_1_/*c*
Protonation site	N1	N1	N7	N7	N7	N7	N9
Type of hydrogen bonding	N—H⋯O, N—H⋯Br, N—H⋯N, O—H⋯O, C—H⋯Br	N—H⋯O, N—H⋯Cl, N—H⋯N, O—H⋯Cl, C—H⋯Cl	N—H⋯O, N—H⋯Br, N—H⋯N, O—H⋯Br, C—H⋯Br	N—H⋯O, N—H⋯Cl, N—H⋯N, O—H⋯Cl, C—H⋯Cl	N—H⋯Cl, N—H⋯O, O—H⋯N, O—H⋯Cl, C—H⋯Cl	N—H⋯Br, N—H⋯O, O—H⋯N, O—H⋯Br, C—H⋯Br	N—H⋯O, N—H⋯Br, O—H⋯O
Type of stacking	–	–	C=O⋯π	C=O⋯π	C=O⋯π	C=O⋯π	C=O⋯π
Primary motif	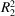 (10)	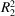 (10)	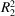 (8)	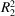 (8)	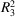 (9)	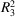 (9)	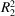 (8)
Secondary motif	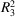 (7), 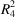 (14)	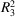 (7), 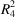 (14)	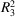 (7), 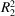 (10), 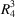 (11)	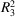 (7), 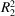 (10), 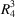 (11)	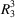 (11), 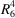 (16), 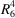 (14)	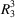 (11), 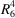 (16), 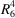 (14)	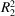 (7), 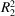 (4)
Type of packing architecture	Ribbon	Ribbon	Ribbon	Ribbon	Wave	Wave	Staircase

**Table 4:** Experimental details

	**I**	**II**
Crystal data		
Chemical formula	C_5_H_5_N_4_O^+^·Br^−^·H_2_O	C_5_H_5_N_4_O_2_^+^·Br^−^·H_2_O
*M*_r_	235.06	251.06
Crystal system, space group	Monoclinic, *P*2_1_/*c*	Monoclinic, *P*2_1_/*c*
Temperature (K)	296	303
*a*, *b*, *c* (Å)	4.8487 (4), 18.4455 (15), 9.0782 (7)	4.9225 (2), 22.7572 (17), 7.5601 (5)
β (°)	94.808 (1)	103.003 (3)
*V* (Å^3^)	809.07 (11)	825.18 (9)
*Z*	4	4
Radiation type	Mo *K*α	Mo *K*α
μ (mm^−1^)	5.05	4.96
Crystal size (mm)	0.46 × 0.26 × 0.21	0.55 × 0.37 × 0.31

Data collection		
Diffractometer	Bruker APEXII CCD	Bruker APEXII CCD
Absorption correction	Multi-scan (*SADABS*; Bruker, 2016 ▸)	Multi-scan (*SADABS*; Bruker, 2016 ▸)
*T*_min_, *T*_max_	0.403, 0.641	0.316, 0.561
No. of measured, independent and observed [*I* > 2σ(*I*)] reflections	17895, 2383, 2037	5810, 1855, 1418
*R*_int_	0.028	0.045
(sin θ/λ)_max_ (Å^−1^)	0.707	0.696

Refinement		
*R*[*F* ^2^ > 2σ(*F* ^2^)], *wR*(*F* ^2^), *S*	0.021, 0.056, 1.05	0.036, 0.080, 1.10
No. of reflections	2383	1855
No. of parameters	128	139
No. of restraints	6	9
H-atom treatment	H atoms treated by a mixture of independent and constrained refinement	Only H-atom coordinates refined
Δρ_max_, Δρ_min_ (e Å^−3^)	0.34, −0.29	0.42, −0.62

Computer programs: *APEX2* and *SAINT* (Bruker, 2016 ▸), *SHELXS97* (Sheldrick 2008 ▸), *SHELXT2014/5* (Sheldrick, 2015*a* ▸), *SHELXL2018/3* (Sheldrick, 2015*b* ▸), *PLATON* (Spek, 2020 ▸), *Mercury* (Macrae *et al.*, 2020 ▸), *POVRay* (Cason, 2004 ▸) and *publCIF* (Westrip,2010 ▸).

## supplementary crystallographic information

### 6-Oxo-6,9-dihydro-1H-purin-7-ium bromide monohydrate (I) Crystal data

**Table:**

C_5_H_5_N_4_O^+^·Br^−^·H_2_O	*F*(000) = 464
*M**_r_* = 235.06	*D*_x_ = 1.930 Mg m^−^^3^
Monoclinic, *P*2_1_/*c*	Mo *K*α radiation, λ = 0.71073 Å
*a* = 4.8487 (4) Å	Cell parameters from 2383 reflections
*b* = 18.4455 (15) Å	θ = 2.2–30.2°
*c* = 9.0782 (7) Å	µ = 5.05 mm^−^^1^
β = 94.808 (1)°	*T* = 296 K
*V* = 809.07 (11) Å^3^	Plate, colourless
*Z* = 4	0.46 × 0.26 × 0.21 mm

### 6-Oxo-6,9-dihydro-1H-purin-7-ium bromide monohydrate (I) Data collection

**Table:**

Bruker APEXII CCD diffractometer	2037 reflections with *I* > 2σ(*I*)
φ and ω scans	*R*_int_ = 0.028
Absorption correction: multi-scan (SADABS; Bruker, 2016)	θ_max_ = 30.2°, θ_min_ = 2.2°
*T*_min_ = 0.403, *T*_max_ = 0.641	*h* = −6→6
17895 measured reflections	*k* = −25→26
2383 independent reflections	*l* = −12→12

### 6-Oxo-6,9-dihydro-1H-purin-7-ium bromide monohydrate (I) Refinement

**Table:**

Refinement on *F*^2^	Secondary atom site location: difference Fourier map
Least-squares matrix: full	Hydrogen site location: mixed
*R*[*F*^2^ > 2σ(*F*^2^)] = 0.021	H atoms treated by a mixture of independent and constrained refinement
*wR*(*F*^2^) = 0.056	*w* = 1/[σ^2^(*F*_o_^2^) + (0.0273*P*)^2^ + 0.2516*P*] where *P* = (*F*_o_^2^ + 2*F*_c_^2^)/3
*S* = 1.05	(Δ/σ)_max_ = 0.002
2383 reflections	Δρ_max_ = 0.34 e Å^−^^3^
128 parameters	Δρ_min_ = −0.29 e Å^−^^3^
6 restraints	Extinction correction: SHELXL2018/3 (Sheldrick 2015b), Fc^*^=kFc[1+0.001xFc^2^λ^3^/sin(2θ)]^-1/4^
Primary atom site location: structure-invariant direct methods	Extinction coefficient: 0.0080 (9)

### 6-Oxo-6,9-dihydro-1H-purin-7-ium bromide monohydrate (I) Special details

**Table:**

Geometry. All esds (except the esd in the dihedral angle between two l.s. planes) are estimated using the full covariance matrix. The cell esds are taken into account individually in the estimation of esds in distances, angles and torsion angles; correlations between esds in cell parameters are only used when they are defined by crystal symmetry. An approximate (isotropic) treatment of cell esds is used for estimating esds involving l.s. planes.

### 6-Oxo-6,9-dihydro-1H-purin-7-ium bromide monohydrate (I) Fractional atomic coordinates and isotropic or equivalent isotropic displacement parameters (Å^2^)

**Table:**

	*x*	*y*	*z*	*U*_iso_*/*U*_eq_	
Br1	−0.30053 (4)	0.47161 (2)	0.77208 (2)	0.04456 (8)	
O6	−0.2120 (2)	0.27474 (6)	0.73221 (11)	0.0337 (2)	
N9	0.4049 (2)	0.18412 (7)	0.42420 (13)	0.0278 (2)	
H9	0.529 (3)	0.1816 (10)	0.3639 (18)	0.041 (5)*	
N3	0.3696 (2)	0.31647 (6)	0.43388 (13)	0.0288 (2)	
N1	0.0413 (3)	0.35239 (6)	0.59830 (14)	0.0293 (2)	
H1	−0.035 (4)	0.3873 (9)	0.638 (2)	0.045 (5)*	
N7	0.1015 (3)	0.15533 (6)	0.57823 (13)	0.0283 (2)	
H7	0.000 (4)	0.1316 (10)	0.6329 (19)	0.044 (5)*	
C5	0.1107 (3)	0.22987 (7)	0.57102 (14)	0.0232 (2)	
C8	0.2792 (3)	0.12926 (8)	0.48894 (16)	0.0309 (3)	
H8	0.312119	0.080303	0.473349	0.037*	
C2	0.2315 (3)	0.36593 (8)	0.50019 (16)	0.0309 (3)	
H2	0.266402	0.414166	0.478442	0.037*	
O1W	−0.7539 (3)	0.60447 (7)	0.73814 (15)	0.0447 (3)	
H1W	−0.653 (4)	0.6346 (10)	0.695 (2)	0.067*	
H2W	−0.671 (4)	0.5639 (7)	0.741 (2)	0.067*	
C4	0.3015 (3)	0.24822 (7)	0.47360 (13)	0.0235 (2)	
C6	−0.0384 (3)	0.28405 (7)	0.64259 (14)	0.0245 (2)	

### 6-Oxo-6,9-dihydro-1H-purin-7-ium bromide monohydrate (I) Atomic displacement parameters (Å^2^)

**Table:**

	*U*^11^	*U*^22^	*U*^33^	*U*^12^	*U*^13^	*U*^23^
Br1	0.05176 (12)	0.02567 (9)	0.06016 (13)	0.00535 (6)	0.02789 (9)	−0.00045 (7)
O6	0.0347 (5)	0.0341 (5)	0.0352 (5)	0.0018 (4)	0.0205 (5)	−0.0004 (4)
N9	0.0272 (5)	0.0312 (6)	0.0267 (5)	0.0032 (4)	0.0122 (5)	−0.0009 (4)
N3	0.0292 (6)	0.0287 (6)	0.0299 (6)	−0.0017 (5)	0.0116 (5)	0.0026 (5)
N1	0.0325 (6)	0.0257 (5)	0.0316 (6)	0.0025 (5)	0.0133 (5)	−0.0016 (5)
N7	0.0311 (6)	0.0251 (5)	0.0301 (6)	−0.0029 (4)	0.0123 (5)	−0.0005 (4)
C5	0.0223 (6)	0.0259 (6)	0.0224 (6)	−0.0010 (5)	0.0072 (5)	−0.0009 (5)
C8	0.0344 (7)	0.0267 (6)	0.0328 (7)	0.0024 (5)	0.0105 (6)	−0.0019 (5)
C2	0.0333 (7)	0.0273 (6)	0.0334 (7)	−0.0011 (5)	0.0103 (6)	0.0033 (5)
O1W	0.0458 (7)	0.0321 (6)	0.0608 (8)	0.0075 (5)	0.0319 (6)	0.0077 (5)
C4	0.0216 (6)	0.0282 (6)	0.0215 (6)	0.0006 (5)	0.0066 (5)	−0.0008 (5)
C6	0.0234 (6)	0.0285 (6)	0.0223 (6)	0.0012 (5)	0.0060 (5)	−0.0014 (5)

### 6-Oxo-6,9-dihydro-1H-purin-7-ium bromide monohydrate (I) Geometric parameters (Å, º)

**Table:**

O6—C6	1.2308 (15)	N7—C8	1.3219 (17)
N9—C8	1.3419 (18)	N7—C5	1.3774 (17)
N9—C4	1.3741 (16)	N7—H7	0.849 (14)
N9—H9	0.847 (14)	C5—C4	1.3748 (17)
N3—C2	1.3078 (18)	C5—C6	1.4221 (17)
N3—C4	1.3579 (16)	C8—H8	0.9300
N1—C2	1.3581 (17)	C2—H2	0.9300
N1—C6	1.3879 (17)	O1W—H1W	0.857 (9)
N1—H1	0.840 (14)	O1W—H2W	0.848 (9)
			
C8—N9—C4	108.32 (10)	N7—C8—N9	109.73 (12)
C8—N9—H9	127.8 (13)	N7—C8—H8	125.1
C4—N9—H9	123.8 (13)	N9—C8—H8	125.1
C2—N3—C4	112.29 (11)	N3—C2—N1	125.15 (13)
C2—N1—C6	125.30 (12)	N3—C2—H2	117.4
C2—N1—H1	119.4 (14)	N1—C2—H2	117.4
C6—N1—H1	115.3 (14)	H1W—O1W—H2W	107.4 (17)
C8—N7—C5	107.98 (11)	N3—C4—N9	127.42 (11)
C8—N7—H7	127.6 (13)	N3—C4—C5	126.22 (12)
C5—N7—H7	124.4 (13)	N9—C4—C5	106.36 (11)
C4—C5—N7	107.61 (11)	O6—C6—N1	122.73 (12)
C4—C5—C6	121.08 (12)	O6—C6—C5	127.31 (13)
N7—C5—C6	131.31 (11)	N1—C6—C5	109.96 (11)
			
C8—N7—C5—C4	−0.02 (16)	N7—C5—C4—N3	179.07 (12)
C8—N7—C5—C6	179.24 (14)	C6—C5—C4—N3	−0.3 (2)
C5—N7—C8—N9	0.27 (17)	N7—C5—C4—N9	−0.23 (15)
C4—N9—C8—N7	−0.42 (17)	C6—C5—C4—N9	−179.58 (12)
C4—N3—C2—N1	0.2 (2)	C2—N1—C6—O6	179.92 (14)
C6—N1—C2—N3	−0.6 (2)	C2—N1—C6—C5	0.5 (2)
C2—N3—C4—N9	179.41 (14)	C4—C5—C6—O6	−179.46 (14)
C2—N3—C4—C5	0.3 (2)	N7—C5—C6—O6	1.4 (3)
C8—N9—C4—N3	−178.90 (14)	C4—C5—C6—N1	−0.12 (18)
C8—N9—C4—C5	0.39 (16)	N7—C5—C6—N1	−179.30 (14)

### 6-Oxo-6,9-dihydro-1H-purin-7-ium bromide monohydrate (I) Hydrogen-bond geometry (Å, º)

**Table:**

*D*—H···*A*	*D*—H	H···*A*	*D*···*A*	*D*—H···*A*
N9—H9···Br1^i^	0.85 (1)	3.08 (2)	3.5397 (12)	117 (2)
N9—H9···O6^i^	0.85 (1)	1.98 (2)	2.7579 (14)	153 (2)
N1—H1···Br1	0.84 (1)	2.41 (1)	3.2419 (12)	170 (2)
N7—H7···O1*W*^ii^	0.85 (1)	1.81 (2)	2.6401 (16)	165 (2)
O1*W*—H1*W*···N3^iii^	0.86 (1)	2.08 (1)	2.9200 (16)	165 (2)
O1*W*—H2*W*···Br1	0.85 (1)	2.48 (1)	3.2894 (12)	161 (2)
C8—H8···Br1^i^	0.93	2.89	3.4875 (15)	123

Symmetry codes: (i) *x*+1, −*y*+1/2, *z*−1/2; (ii) −*x*−1, *y*−1/2, −*z*+3/2; (iii) −*x*, −*y*+1, −*z*+1.

### 2,6-Dioxo-2,3,6,9-tetrahydro-1H-purin-7-ium bromide monohydrate (II) Crystal data

**Table:**

C_5_H_5_N_4_O_2_^+^·Br^−^·H_2_O	*F*(000) = 496
*M**_r_* = 251.06	*D*_x_ = 2.021 Mg m^−^^3^
Monoclinic, *P*2_1_/*c*	Mo *K*α radiation, λ = 0.71073 Å
*a* = 4.9225 (2) Å	Cell parameters from 1418 reflections
*b* = 22.7572 (17) Å	θ = 2.9–29.6°
*c* = 7.5601 (5) Å	µ = 4.96 mm^−^^1^
β = 103.003 (3)°	*T* = 303 K
*V* = 825.18 (9) Å^3^	Plate, colourless
*Z* = 4	0.55 × 0.37 × 0.31 mm

### 2,6-Dioxo-2,3,6,9-tetrahydro-1H-purin-7-ium bromide monohydrate (II) Data collection

**Table:**

Bruker APEXII CCD diffractometer	1418 reflections with *I* > 2σ(*I*)
φ and ω scans	*R*_int_ = 0.045
Absorption correction: multi-scan (SADABS; Bruker, 2016)	θ_max_ = 29.7°, θ_min_ = 2.9°
*T*_min_ = 0.316, *T*_max_ = 0.561	*h* = −6→6
5810 measured reflections	*k* = −30→30
1855 independent reflections	*l* = −9→9

### 2,6-Dioxo-2,3,6,9-tetrahydro-1H-purin-7-ium bromide monohydrate (II) Refinement

**Table:**

Refinement on *F*^2^	Primary atom site location: structure-invariant direct methods
Least-squares matrix: full	Secondary atom site location: difference Fourier map
*R*[*F*^2^ > 2σ(*F*^2^)] = 0.036	Hydrogen site location: difference Fourier map
*wR*(*F*^2^) = 0.080	Only H-atom coordinates refined
*S* = 1.10	*w* = 1/[σ^2^(*F*_o_^2^) + (0.0151*P*)^2^ + 1.7175*P*] where *P* = (*F*_o_^2^ + 2*F*_c_^2^)/3
1855 reflections	(Δ/σ)_max_ < 0.001
139 parameters	Δρ_max_ = 0.42 e Å^−^^3^
9 restraints	Δρ_min_ = −0.62 e Å^−^^3^

### 2,6-Dioxo-2,3,6,9-tetrahydro-1H-purin-7-ium bromide monohydrate (II) Special details

**Table:**

Geometry. All esds (except the esd in the dihedral angle between two l.s. planes) are estimated using the full covariance matrix. The cell esds are taken into account individually in the estimation of esds in distances, angles and torsion angles; correlations between esds in cell parameters are only used when they are defined by crystal symmetry. An approximate (isotropic) treatment of cell esds is used for estimating esds involving l.s. planes.

### 2,6-Dioxo-2,3,6,9-tetrahydro-1H-purin-7-ium bromide monohydrate (II) Fractional atomic coordinates and isotropic or equivalent isotropic displacement parameters (Å^2^)

**Table:**

	*x*	*y*	*z*	*U*_iso_*/*U*_eq_	
Br1	−0.16454 (8)	0.20412 (2)	0.47569 (6)	0.03200 (14)	
O6	0.8033 (5)	0.47823 (12)	0.6033 (4)	0.0318 (6)	
C6	0.6301 (7)	0.44615 (16)	0.6465 (5)	0.0241 (8)	
N1	0.5267 (7)	0.45642 (14)	0.7988 (4)	0.0273 (7)	
H1	0.578 (8)	0.4867 (13)	0.855 (5)	0.033*	
C2	0.3313 (8)	0.42399 (16)	0.8628 (5)	0.0254 (8)	
O2	0.2541 (6)	0.43894 (13)	0.9992 (4)	0.0380 (7)	
N3	0.2278 (6)	0.37489 (13)	0.7647 (4)	0.0251 (7)	
H3	0.126 (7)	0.3545 (16)	0.812 (5)	0.030*	
C4	0.3212 (7)	0.36166 (15)	0.6147 (5)	0.0238 (8)	
C5	0.5108 (7)	0.39456 (16)	0.5538 (5)	0.0237 (8)	
N7	0.5509 (7)	0.36852 (14)	0.3972 (4)	0.0273 (7)	
H7	0.651 (8)	0.3804 (18)	0.332 (5)	0.033*	
C8	0.3919 (8)	0.32168 (18)	0.3652 (6)	0.0304 (9)	
H8	0.384 (9)	0.2947 (18)	0.265 (6)	0.036*	
N9	0.2477 (7)	0.31597 (14)	0.4956 (5)	0.0279 (7)	
H9	0.148 (7)	0.2876 (14)	0.503 (6)	0.033*	
O1W	0.8947 (7)	0.42353 (14)	0.2374 (4)	0.0434 (8)	
H1WA	0.983 (9)	0.4523 (16)	0.298 (6)	0.065*	
H1WB	0.980 (9)	0.415 (2)	0.154 (5)	0.065*	

### 2,6-Dioxo-2,3,6,9-tetrahydro-1H-purin-7-ium bromide monohydrate (II) Atomic displacement parameters (Å^2^)

**Table:**

	*U*^11^	*U*^22^	*U*^33^	*U*^12^	*U*^13^	*U*^23^
Br1	0.0307 (2)	0.0253 (2)	0.0413 (2)	−0.00152 (17)	0.01087 (16)	−0.00286 (18)
O6	0.0317 (15)	0.0309 (15)	0.0360 (16)	−0.0110 (12)	0.0147 (13)	−0.0028 (12)
C6	0.0231 (18)	0.0217 (18)	0.028 (2)	−0.0001 (14)	0.0057 (16)	0.0046 (15)
N1	0.0294 (17)	0.0253 (17)	0.0292 (19)	−0.0097 (14)	0.0111 (15)	−0.0046 (14)
C2	0.0255 (19)	0.0224 (19)	0.029 (2)	−0.0030 (15)	0.0079 (17)	0.0042 (16)
O2	0.0431 (17)	0.0432 (18)	0.0338 (17)	−0.0118 (14)	0.0216 (14)	−0.0079 (14)
N3	0.0246 (16)	0.0232 (16)	0.0305 (18)	−0.0041 (12)	0.0128 (14)	0.0050 (13)
C4	0.0236 (18)	0.0193 (17)	0.027 (2)	0.0011 (14)	0.0028 (15)	0.0039 (15)
C5	0.0231 (18)	0.0246 (18)	0.0232 (19)	−0.0018 (14)	0.0050 (15)	−0.0008 (15)
N7	0.0291 (17)	0.0286 (17)	0.0265 (18)	−0.0023 (14)	0.0109 (14)	0.0005 (14)
C8	0.035 (2)	0.028 (2)	0.028 (2)	−0.0013 (17)	0.0051 (18)	−0.0048 (17)
N9	0.0299 (17)	0.0186 (15)	0.0346 (19)	−0.0040 (13)	0.0061 (15)	0.0000 (14)
O1W	0.0484 (19)	0.0449 (19)	0.046 (2)	−0.0199 (15)	0.0290 (16)	−0.0172 (15)

### 2,6-Dioxo-2,3,6,9-tetrahydro-1H-purin-7-ium bromide monohydrate (II) Geometric parameters (Å, º)

**Table:**

O6—C6	1.221 (4)	C4—N9	1.370 (5)
C6—N1	1.380 (5)	C5—N7	1.378 (5)
C6—C5	1.425 (5)	N7—C8	1.312 (5)
N1—C2	1.383 (5)	N7—H7	0.82 (2)
N1—H1	0.82 (2)	C8—N9	1.344 (5)
C2—O2	1.224 (5)	C8—H8	0.97 (4)
C2—N3	1.374 (5)	N9—H9	0.82 (2)
N3—C4	1.350 (5)	O1W—H1WA	0.857 (10)
N3—H3	0.82 (2)	O1W—H1WB	0.860 (10)
C4—C5	1.355 (5)		
			
O6—C6—N1	122.2 (3)	C5—C4—N9	107.2 (3)
O6—C6—C5	126.6 (4)	C4—C5—N7	107.3 (3)
N1—C6—C5	111.2 (3)	C4—C5—C6	121.8 (3)
C6—N1—C2	128.1 (3)	N7—C5—C6	130.9 (3)
C6—N1—H1	116 (3)	C8—N7—C5	108.2 (3)
C2—N1—H1	115 (3)	C8—N7—H7	125 (3)
O2—C2—N3	122.2 (3)	C5—N7—H7	127 (3)
O2—C2—N1	121.2 (3)	N7—C8—N9	109.6 (4)
N3—C2—N1	116.6 (3)	N7—C8—H8	125 (3)
C4—N3—C2	118.7 (3)	N9—C8—H8	125 (3)
C4—N3—H3	126 (3)	C8—N9—C4	107.7 (3)
C2—N3—H3	115 (3)	C8—N9—H9	123 (3)
N3—C4—C5	123.6 (3)	C4—N9—H9	129 (3)
N3—C4—N9	129.2 (3)	H1WA—O1W—H1WB	107 (2)
			
O6—C6—N1—C2	179.8 (4)	N9—C4—C5—C6	179.6 (3)
C5—C6—N1—C2	−0.7 (5)	O6—C6—C5—C4	179.3 (4)
C6—N1—C2—O2	−178.6 (4)	N1—C6—C5—C4	−0.1 (5)
C6—N1—C2—N3	0.8 (6)	O6—C6—C5—N7	−1.2 (7)
O2—C2—N3—C4	179.3 (4)	N1—C6—C5—N7	179.3 (4)
N1—C2—N3—C4	−0.1 (5)	C4—C5—N7—C8	−0.1 (4)
C2—N3—C4—C5	−0.6 (5)	C6—C5—N7—C8	−179.6 (4)
C2—N3—C4—N9	−179.3 (4)	C5—N7—C8—N9	0.1 (5)
N3—C4—C5—N7	−178.8 (3)	N7—C8—N9—C4	0.0 (4)
N9—C4—C5—N7	0.1 (4)	N3—C4—N9—C8	178.7 (4)
N3—C4—C5—C6	0.8 (6)	C5—C4—N9—C8	−0.1 (4)

### 2,6-Dioxo-2,3,6,9-tetrahydro-1H-purin-7-ium bromide monohydrate (II) Hydrogen-bond geometry (Å, º)

**Table:**

*D*—H···*A*	*D*—H	H···*A*	*D*···*A*	*D*—H···*A*
N1—H1···O2^i^	0.82 (2)	2.09 (2)	2.903 (4)	175 (4)
N3—H3···Br1^ii^	0.82 (2)	2.48 (2)	3.301 (3)	176 (4)
N7—H7···O1*W*	0.82 (2)	1.81 (2)	2.609 (4)	163 (4)
N9—H9···Br1	0.82 (2)	2.43 (2)	3.237 (3)	172 (4)
O1*W*—H1*WA*···O6^iii^	0.86 (1)	1.95 (1)	2.802 (4)	171 (5)
O1*W*—H1*WB*···Br1^iv^	0.86 (1)	3.03 (4)	3.490 (3)	115 (3)
O1*W*—H1*WB*···O2^v^	0.86 (1)	2.05 (3)	2.816 (4)	149 (4)

Symmetry codes: (i) −*x*+1, −*y*+1, −*z*+2; (ii) *x*, −*y*+1/2, *z*+1/2; (iii) −*x*+2, −*y*+1, −*z*+1; (iv) *x*+1, −*y*+1/2, *z*−1/2; (v) *x*+1, *y*, *z*−1.

### Percentage of non-covalent interaction in supramolecular packing analyzed by Hirshfeld surface analysis

**Table:**

		
CONTACT	SALT (I)	SALT (II)
H···Br /Br···H	22.3%	25.4%
O···H/H···O	19.7%	23.4%
H···N/N···H	13.5%	7.5%
C···H/H···C	6.4%	9.6%
H···H	23.4%	15.9%
